# Supplementary material for: Species independence of eye lens dimensions in teleosts and elasmobranchs
Source: PLoS One. 2023 Jun 1;18(6):e0286388. doi: 10.1371/journal.pone.0286388 (PMC10234530; doi:10.1371/journal.pone.0286388)
Supplement: Attachment — Submitted filename: response to reviewers of Leifsdottir and Campana (1).docx [file pone.0286388.s002.docx]

**Response to Reviewers of PONE-D-22-23795 - Species independence of eye lens dimensions in teleost and elasmobranchs**

We thank the reviewer for his many helpful comments. In the following, we have put the reviewer’s comments in italics while our response is in blue font.

*Reviewer #1: ### Introduction

The authors start with a summary of latest interest regarding vertebrate eye lenses, after which they outline the lens development and an overall structure of a lens. They expand that topic describing three, significant to their research, regions of a fish eye lens: outer cortex, hard core, and embryonic region. This section could use a clarification. On the first pass I misunderstood that the lens capsule was one of the three regions. I suggest either 1) enumerating numerically or textually the regions as they are listed in text (as in this very line) or 2) listing all three regions before discussing them. The authors describe an issue with inconsistency regarding naming in the literature, defining clearly what terms will they use for each region of interest (Hardened lens and central hardened lens, HL and CHL respectively).*
The description of the three lens regions has been clarified (enumerated) as suggested.

*The next section the authors start strong claiming HL plays central role in maintaining focused vision. I cannot agree with that statement. HL’s role is as important as the rest of the lens in forming image. In the study the authors refer to (Schartau et al. 2009), the changes in optical properties happen pass the cortex-HL horizon at 70% radius (R) demonstrating that not only the HL plays an important role. The authors pose a question of what the function of such a well-defined cortex-HL horizon is. They suggest that ubiquity of horizon’s location points to optical properties and put in the opposition the transparency caused by the loss of intracellular structures. I am not sure why those two were selected as opposing hypotheses since the intracellular loss happens way pass the 70% R all the way to 92% R. This makes the work’s motivation somewhat weak. Despite that, the authors close the section with clearly defined study objectives: testing for relative location of cortex-HL horizon in different species.*
This is one of the most important issues identified by the reviewer, so it is the issue to which we have given the most attention in the revision. We have tried to keep the wording general in the Introduction, focusing on what others have reported, not necessarily what we believe. However, in the Discussion, we have revised extensively, and provided considerable support for the reviewer’s interpretation.

*### Materials and methods

The authors provide a detailed description of samples collection: time, location, conditions, number of individuals, and their species. They describe the methodology regarding euthanasia, extraction of lens from a fish eye, extraction of HL from a lens, and measurements of fishes, lenses, and HL. Materials and methods section is solid and detailed. However, “error in identifying the horizon (…) was virtually impossible” has no place in scientific publication. Errors can be highly unlikely but never impossible, especially when the used method involves squashing a gelatinous sphere with fingers. This was particularly surprising to read because the reminder of this sentence describes the best possible approach to the problem: measuring lenses from both eyes and comparing them. Which is exactly what the authors did.*

Agreed – this section has been reworded. To provide context for the original wording, a previous reviewer had argued that our method could not possibly identify a well-defined core. Clearly, we over-compensated in our original wording.

*### Results

Firstly, the authors report that lens diameter (LD) and HL diameter (HD) are linearly correlated with total length (TL) of the fish, but the relation is different for different species. The relation between LD and HD is also linear. There is no significant difference between individual species, only when two groups (teleost vs elasmobranchs) were compared. This is likely caused by thorny skate which stands out from the rest. The authors establish combined for all species HD:LD ratio to be 69%. They also provide a breakdown of ratios for both groups (68% and 70%). The ratio is also invariant to TL. The authors back all their findings with statistical analysis. At the end the authors include data from five lenses of species not involved in this study. They find no correlation between DH and fork length, but they point out small sample size and sample size range. This part brings nothing to the study and makes me wonder why mentioning this at all.*
Agreed. The results reporting the five lenses of Greenland shark have been removed.

*\### Discussion

The authors discuss that the link between LD and HL agrees with other studies and that HL increases its size to keep the pace with the fish size. The ratio of HD:LD is 69% which also agrees with reports on other species. The authors argue that whereas the relation between TL and LD is different for different species, consistent HD:LD ratio suggest HL increasing size is linked to maintenance of optical quality during the lens growth. Further the authors explain that refractive index gradient (decreasing from the center to the surface) present in most vertebrate eyes increases the optical power and reduces spherical aberration. The refractive index gradient is especially important to spherical lenses (such as in fishes) due to surrounding materials of similar refractive index (water, cornea, intraocular vitreous humor).

Unfortunately, in the next part the authors bring up outdated views regarding the refractive index profile in a fish eye lens. They refer to conclusions drawn by Fernald and Wright (1983) that the HL has a uniform refractive index, that optical properties are maintained during the growth by maintaining the cortex-HL horizon, and that central fibers are compressed due to high refractive index. Continuous gradient of refractive index has been demonstrated in Kröger 2013. A study that involved Fernald himself (Kröger et al. 2001, DOI: 10.1016/s0042-6989(00)00283-2) showed that optical properties are maintained during the growth by maintaining the gradient profile normalized to radius. The compression of fibers has also been disproved relatively recently (Kozłowski and Kröger 2019, DOI: 10.1016/j.visres.2019.06.008). The authors comment on Fernald and Wright’s conclusions by mentioning early studies that disagreed with Fernald and Wright but, as publications I brought up, demonstrate it is far from a debate. The section is closed with a conclusion that HD:LD ratio similar between species is important for maintenance of optical quality.*
Agreed. The Discussion has been extensively reworded to bring in the most recent literature, including the papers cited above.

*The authors continue with a summary of lens development leading to the fact that fiber cells lose their organelles and nuclei. They also mention that cells lose the water which is not the case. The authors seem surprised that organelle free zone (OFZ) is reported up to 92% R whereas the HL is only up to 70% R. Presence of water is exactly the reasons. It would be impossible to rub off the gelatinous outer cortex if loss of organelles would go with a loss of water. The authors also believe that 70% R reported by Schartau et al. 2009 is a threshold of optical plasticity. In Schartau’s work one can observe small differences at regions as close as 20% R (Day vs Night). If those differences are too small to be convincing, Jönsson et al. 2014 (DOI: 10.1007/s00359-014-0941-z) showed changes in lenes of Atlantic cod along almost the entire radius below the 70% R. Naturally, based on those wrong assumptions the authors build an incorrect conclusion that optical plasticity is better reflected by the HL rather than by the OFZ, where studies shows neither seem to really affect it.*
Agreed. The Discussion has been extensively revised to reflect current understanding of refractive index gradient profiles. We have retained the reporting of Schartau’s 70% threshold, since its similarity to our HD:LD ratio is too close to be a coincidence. However, we no longer frame this in terms of a static thresold.

*The discussion is closed with a possible application of back calculations for estimates of fish sizes based on the size of isolated central core.

\### Final remarks
I know my review will not make the authors’ day particularly good, but I believe that the experiment was done and reported well, and the findings can contribute to our collective knowledge on lenses. The science is good but it is not a publication material in its current form. The study needs a better motivation and conclusions drawn with better understanding of relation between optics, refractive index, and proteins.*
In fact, the reviewer’s comments were extremely helpful. We are much happier with the resulting revision.

*When looking at single species, fixed cortex-HL horizon means similar density of proteins, which in turn means refractive index. The horizon must be maintained in that position throughout the growth because the entire refractive index profile must remain the same. It is very interesting that one specific refractive index (thus protein concentration, thus hardness) always ends up in the same relative position for different species, suggesting that they must have a very similar refractive index profile.*

This was a useful comment, and we have incorporated it into both the Discussion and the Abstract.
