# Supplementary material for: Species independence of eye lens dimensions in teleosts and elasmobranchs
Source: PLoS One. 2023 Jun 1;18(6):e0286388. doi: 10.1371/journal.pone.0286388 (PMC10234530; doi:10.1371/journal.pone.0286388)
Supplement: Attachment — Submitted filename: Response to Reviewers.docx [file pone.0286388.s003.docx]

**Response to Reviewers of PONE-D-22-23795R1 - Species independence of eye lens dimensions in teleost and elasmobranchs**

We thank the reviewer for his helpful comments. In the following, we have put the reviewer’s comments in italics while our response is in blue font.

*Reviewer #1: The authors addressed all the concerns from the original review. I am returning with one minor remark, few mistakes, and a comment. I will discuss them as they appear in text:

Line 79: "...a refractive index profile (...) which are common" - I think it should be "is"*
The wording has been changed, “are” has been corrected to “is”.
 *Lines 167-175: The way it is formulated suggests a direct contradiction. Both Kröger and Wang talk about optical properties with lens growth - constant vs changing, but both mean the "lens growth" in a different way. Wang looked at the full growth from larva to adult, whereas Kröger looked at individuals form sexual maturity up to 3 years. Wang's results for similar range show that the changes are very small (117dpf - 880dpf). I leave the clarification at the authors' discretion.*
Agree, we have changed the wording slightly so that it does not suggest a direct contradiction.
 *Lines 172-173: "...continuous and declining refractive gradient from the surface towards a plateau near the center of the lens" - Refractive index is the highest in the center, so it should be "increasing" instead of "declining". Alternatively: "...declining refractive gradient from a plateau near the center of the lens towards the surface"*
The wording has been changed, “declining” has been corrected to “increasing”. *Line 206: "likely in exchange with water" - This part can be removed. The mechanism we suspected was transport of proteins which would dissolve in water rather than exchange it.*
The part “likely in exchange with water” has been removed. *Line 177: "both reported a uniform refractive index at the center of the fish eye lens" - I have been thinking about this for a very long time. I disagree with this statement but I have trouble accusing the authors of being incorrect. To me Wang's plateau is not a region of constant refractive index, but rather a region in which the change is very small. However, what does it mean "constant"? What differentiate "constant" from "plateau" by my definition, would be a difference in refractive index several places after comma. Others may look at this with different precision or threshold. As such it is more of an opinion rather than a fact, making it an academic discussion which is outside the scope of a review. Nevertheless I am bringing authors' attention to this detail.*
We agree with the reviewer that the difference of “constant” and “plateau” could be an academic discussion. However, we have a different understanding of Wang’s plateau and would like to keep the original wording.
